# Longitudinal Dopamine D2 Receptor Changes and Cerebrovascular Health in Aging

**DOI:** 10.1212/WNL.0000000000200891

**Published:** 2022-09-20

**Authors:** Nina Karalija, Jarkko Johansson, Goran Papenberg, Anders Wåhlin, Alireza Salami, Ylva Köhncke, Andreas M. Brandmaier, Micael Andersson, Jan Axelsson, Katrine Riklund, Martin Lövdén, Ulman Lindenberger, Lars Bäckman, Lars Nyberg

**Affiliations:** From the Departments of Radiation Sciences, Diagnostic Radiology (N.K., J.J., K.R., L.N.) and Radiation Physics (A.W., J.A.), Department of Applied Physics and Electronics (A.W.), and Umeå Center for Functional Brain Imaging (UFBI) (N.K., J.J., A.W., A.S., M.A., J.A., K.R., L.N.), Umeå University; Aging Research Center (G.P., A.S., L.B.), Karolinska Institutet & Stockholm University; Department of Integrative Medical Biology (A.S., M.A., L.N.), and Wallenberg Center for Molecular Medicine (A.S., L.N.), Umeå University, Sweden; Center for Lifespan Psychology (Y.K., A.M.B., U.L.), Max Planck Institute for Human Development; Max Planck UCL Centre for Computational Psychiatry and Ageing Research (A.M.B., U.L.), Berlin, Germany and London, UK; and Department of Psychology (M.L.), University of Gothenburg, Sweden.

## Abstract

**Background and Objectives:**

Cross-sectional studies suggest marked dopamine (DA) decline in aging, but longitudinal evidence is lacking. The aim of this study was to estimate within-person decline rates for DA D2-like receptors (DRD2) in aging and examine factors that may contribute to individual differences in DRD2 decline rates.

**Methods:**

We investigated 5-year within-person changes in DRD2 availability in a sample of older adults. At both occasions, PET with ^11^C-raclopride and MRI were used to measure DRD2 availability in conjunction with structural and vascular brain integrity.

**Results:**

Longitudinal analyses of the sample (baseline: n = 181, ages: 64–68 years, 100 men and 81 women; 5-year follow-up: n = 129, 69 men and 60 women) revealed aging-related striatal and extrastriatal DRD2 decline, along with marked individual differences in rates of change. Notably, the magnitude of striatal DRD2 decline was ∼50% of past cross-sectional estimates, suggesting that the DRD2 decline rate has been overestimated in past cross-sectional studies. Significant DRD2 reductions were also observed in select extrastriatal regions, including hippocampus, orbitofrontal cortex (OFC), and anterior cingulate cortex (ACC). Distinct profiles of correlated DRD2 changes were found across several associative regions (ACC, dorsal striatum, and hippocampus) and in the reward circuit (nucleus accumbens and OFC). DRD2 losses in associative regions were associated with white matter lesion progression, whereas DRD2 losses in limbic regions were related to reduced cortical perfusion.

**Discussion:**

These findings provide the first longitudinal evidence for individual and region-specific differences of DRD2 decline in older age and support the hypothesis that cerebrovascular factors are linked to age-related dopaminergic decline.

Neurocognitive impairment compromises health for many older adults. Owing to a globally aging population, efforts are directed at identifying modifiable brain changes within key pathologic aging routes.^1,2^ In this context, the age sensitivity of the dopamine (DA) pathways has been highlighted in various studies.^3^ Cross-sectional studies suggest that the availability of DA receptors and transporters is reduced by 8%–14% per decade across the lifespan.^4^ Most studies assessed DA D2-like receptor (DRD2) availability because of their central role for cognition and in neurodegenerative conditions.^3,5^

A major shortcoming is that most human in vivo DA studies to date relied on cross-sectional designs with small samples (median: 21 participants).^4^ Cohort effects in cross-sectional studies can result in overestimation or underestimation of the extent of within-person brain and behavioral changes.^6-8^ Accordingly, findings from cross-sectional studies may diverge from longitudinal results in patterns of brain-behavior change-change associations.^9^ The few existing longitudinal DRD2 studies were conducted with a few days or months between sessions and revealed little change.^10,11^ Longitudinal assessments over longer periods are key to understand the magnitude of individual differences in DA losses and to delineate factors that contribute to DA losses in aging.^12^

Vascular changes occur early in the adult lifespan, are part of dementia pathologies,^13,14^ and may modulate DA integrity. Ischemic strokes are followed by DA changes,^15^ and Parkinson disease is associated with an elevated risk of cardiovascular disease.^16^ Associations among markers of cerebral small vessel disease and DA integrity have also been observed in healthy older samples.^17^ Hence, maintained vascular health may constitute one predictor of preserved DA integrity in aging, but this relation has not been probed using longitudinal data.

We present the first long-term longitudinal investigation of aging-related DRD2 changes, with data from the Cognition, Brain, and Aging (COBRA) study.^18^ In COBRA, healthy adults (>60 years) have undergone PET with ^11^C-raclopride, MRI, and assessment of health, lifestyle, and cognition at 2 occasions separated by 5 years. The relatively large sample size (n = 181 and 129 at baseline and follow-up) permits analyses of trends for DRD2 change across regions, individual differences in change, and associations between DRD2 and other brain changes.

First, we assessed longitudinal rates of DRD2 change to corroborate findings of aging-related DRD2 differences in cross-sectional studies (∼8% per decade in the striatum^4^). Second, we investigated whether the regional DRD2 decline rates correlated within striatal compartments and across the mesolimbic and mesocortical DA pathways.^19,20^ Finally, we tested whether individual differences in DRD2 decline were related to reduced cerebrovascular health, using white matter (WM) lesion progression and reductions in cerebral perfusion as indicators of vascular health.^17^ We expected the vascular-DRD2 link to be strongest for the hippocampus and basal ganglia because of these regions' sensitivity to vascular insult.^21,22^

## Methods

### Standard Protocol Approvals, Registrations, and Patient Consents

This study was approved by the Swedish Ethical Review Authority (Umeå, Sweden; registration number: 2012-57-31M) and conducted in accordance with the Declaration of Helsinki. Written informed consent was obtained from all participants before any testing.

### Sample

The original sample consisted of 181 healthy older adults (100 men, 81 women; ages: 64–68 years, mean: 66.2 ± 1.2) randomly selected from the population registry in Umeå (in northern Sweden) who were offered participation. Eligible participants were free from disorders and conditions that can alter brain and cognitive functioning. Exclusion criteria were neurologic and psychiatric disorders, epilepsy, previous brain trauma, intellectual disability, a Mini-Mental State Examination (MMSE) score of <27, structural brain abnormalities (inspection performed by neuroradiologists), cancer, diabetes, severe auditory and visual impairments, claustrophobia, and metal implants. The assessment battery included brain imaging with ^11^C-raclopride-PET and MRI, cognitive testing, and collection of demographics, health, and lifestyle data and blood samples. The baseline sample, assessment battery, and exclusion criteria, and details for the a priori statistical power analyses, have been described previously.^18^ The statistical power analyses revealed that for a sample size of 180, a 5-year separation between test waves, and an estimated attrition rate of 20%, the expected power to detect significant individual differences in change over 5 years was 83% for the cognitive measures (which were deemed to have lower reliability than the brain measures). The attrition rate was based on longitudinal data for 60–70-year-old adults from a separate study.^8^ All participants were offered to undergo the assessments for a second time at the 5-year follow-up, when 129 returned (69 men, 60 women; ages: 69–73 years, mean: 71.2 ± 1.2). The duration between test waves was 60.1 ± 0.6 months for PET and 60.0 ± 0.4 months for MRI. The scheduled time between MRI and PET was 2 days, but technical issues, traveling, and disease caused longer duration between sessions for some (baseline: mean = 3.9 ± 5.7 days, max: 44 days; follow-up: mean = 3.8 ± 7.6 days, max: 72 days).

### Brain Imaging

MRI was performed with a 3 tesla Discovery MR 750 scanner (General Electric, Milwaukee, WI), and PET data were acquired with a Discovery PET/CT 690 (General Electric) at both time points.

### Regional Volumes

T1-weighted images were obtained with echo time 3.2 milliseconds, flip angle 12°, repetition time 8.19 milliseconds, 176 slices with thickness 1.0 mm, and field of view 25.0 cm with resolution 0.98 mm upsampled to 0.49 mm. The longitudinal image processing pipeline in FreeSurfer, version 6.0, was used to process T1-weighted images and derive estimates of gray matter (GM), WM, and lateral ventricle size. Subcortical GM segmentations and cortical parcellations^23^ (see eMethods, links.lww.com/WNL/C161) were used to define regions of interest (ROIs) for DRD2 and perfusion assessment.

### DRD2 Availability

A 55-minute, 18-frame dynamic PET scan was acquired during rest after IV bolus injection of approximately 250 MBq ^11^C-raclopride (baseline: 263.5 ± 19.0 MBq; follow-up: 260.2 ± 15.0 MBq; *t*(123) = 1.8; *p* = 0.076). The range for the injected mass of raclopride was larger at baseline (0.17–9.48 μg) than at follow-up (0.11 and 2.67 μg; *t*(124) = 4.6; *p* < 0.001). An attenuation CT scan (20 mA, 120 kV, 0.8 seconds/revolution) preceded ligand injection. Attenuation- and decay-corrected images (47 slices, field of view = 25 cm, 256 × 256-pixel transaxial images, voxel size = 0.977 × 0.977 × 3.27 mm^3^) were reconstructed with the iterative algorithm VUE Point HD-SharpIR (GE; 6 iterations, 24 subsets, 3.0 mm postfiltering; full width at half maximum: 3.2 mm). PET images were motion corrected and coregistered with the structural T1-weighted images from the corresponding session (baseline and follow-up) using the Statistical Parametric Mapping software (SPM12). As a source for coregistration, the mean of the first 5 frames was used. PET images from both time points were coregistered with the baseline T1 image for 3 participants (no MRI at follow-up). Two individuals declined to undergo PET at follow-up.

DRD2 binding potential (BP_ND_) was estimated with and without correction for partial volume effects (PVEs). Regional PVE correction was conducted using the symmetric geometric transfer matrix implemented in FreeSurfer.^24^ An incremental PVE-correction approach was used in which (1) the initial correction was achieved using resolution modeling in the iterative image reconstruction procedure (SHARP-IR), and (2) the remnant PVE was controlled for using the ROI-based geometric transfer matrix approach. The size of the secondary correction kernel was estimated empirically (point spread function of 2.5 mm; isotropic) to achieve a similar level of correction as earlier.^25^ FreeSurfer segmentations and preprocessed PET data were used to estimate PVE-corrected regional radioactivity concentrations per ROI and time frame. PVE-corrected BP_ND_ estimates were calculated with the multilinear reference tissue model (MRTM) on dynamic PVE-corrected data, with cerebellar GM radioactivity as an indirect input function.

^11^C-raclopride BP_ND_ values without PVE correction were estimated using Logan analysis^26^ from time activity curves within T1-segmented ROIs (median of ROI voxel values from time frames between 18 and 55 minutes). The cerebellar GM served as the reference area. The 2 pipelines (MRTM vs Logan) allowed for comparisons of the robustness of DRD2 decline patterns (Table 1). The analyses that appear in Results were conducted with PVE-corrected data. Notably, analyses were replicated with BP_ND_ values without PVE correction, including assessments of change over time (Table 1, and for a more extensive set of regions in eTable 2, links.lww.com/WNL/C161), associations for DRD2 changes across regions (eTable 1), and DRD2-GM volume associations (eTable 3).

**Table 1:**
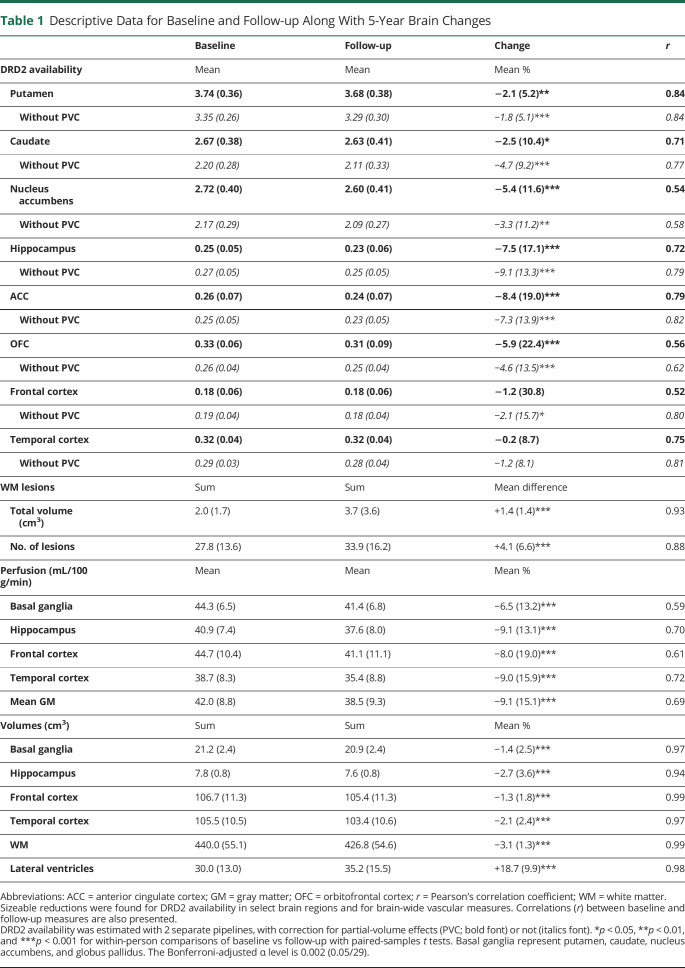
Descriptive Data for Baseline and Follow-up Along With 5-Year Brain Changes

### Perfusion

Three-dimensional pseudocontinuous arterial spin labeling (pcASL) was acquired with background suppression and spiral readout. Labeling time = 1.5 seconds, postlabeling delay time = 1.5 seconds, field of view = 24 cm, slice thickness = 4 mm, and acquisition resolution = 8 × 512 (arms × data points), with the number of averages set at 3. The total scanning time was approximately 5 minutes. This sequence provided whole-brain perfusion in mL/100 g/min. The reconstructed voxel size was 1.875 × 1.875 × 4 mm^3^. Quantitative perfusion maps were calculated using a postprocessing tool installed on the scanner by the manufacturer. Mean GM perfusion was computed for FreeSurfer-segmented ROIs as the average of the individual perfusion estimates weighted by volume.

### WM Lesions

WM hyperintensities were segmented from a fluid-attenuated inversion recovery image (48 slices, slice thickness = 3 mm, TE = 120 milliseconds, TR = 8,000 milliseconds, and field of view = 24 × 24 cm) with the lesion growth algorithm,^27^ as implemented in the LST toolbox version 2.0.14 for SPM12^28^ (eMethods, links.lww.com/WNL/C161).

### Cognition

Participants performed the MMSE, in which a minimum of 27 points (max: 30 points) was required for inclusion at baseline. A digit-symbol coding subtest from the Wechsler Adult Intelligence Scale was also administered in which participants received 1 point per correct item coding (duration: 90 seconds). Semantic knowledge was assessed with a vocabulary test. For each word, 1 correct synonym of 5 possible alternatives was to be selected (30 words; 1 point per correct answer).

### Lifestyle and Health

Hours per week spent on various social, intellectual, and physical activities were assessed through self-report questionnaires. History of known disorders, medicine intake, and nicotine consumption was documented. Blood pressure was measured in a sitting position. The body mass index (BMI) was calculated from height and weight. Ten-year cardiovascular disease risk (%) was calculated from hypertension and diabetes diagnosis, systolic blood pressure, BMI, smoking, age, and sex, according to an established model.^17^ ApoE ε4 allele status was assessed from blood samples.^29^

### Statistical Analyses

Analyses were performed with SPSS (version 26) and Ωnyx.^30,31^ Descriptive data are presented with mean or sum (over the left and right hemispheres for brain data), SDs, or frequencies. The significance level for all tests was 5%.

Univariate outliers were defined as >3.29 SD from the mean and excluded as pairwise deletions per ROI and modality. For WM lesions, this procedure was performed twice because of the remaining outliers after the first round of exclusions. Multivariate outliers across ROIs per brain domain (i.e., ROIs listed in eTable 2, links.lww.com/WNL/C161) were identified according to the Mahalanobis distance (*p* < 0.001) and excluded as listwise deletions in analyses of baseline (n = 0 for DRD2 data; n = 1 for volumes; n = 1 for perfusion), follow-up (n = 0 for DRD2; n = 2 for volumes; n = 1 for perfusion), and change (n = 2 for perfusion; n = 7 for DRD2; and n = 0 for volumes). The effective sample for analyses ranged between 120 and 129 depending on the ROI and modality at baseline and follow-up and between 111 and 126 for estimates of change. After exclusions, values for DRD2s, volumes, and perfusion and the number of WM lesions were normally distributed (skewness: −0.73 to 0.90; kurtosis: −0.68 to 1.67) and slightly skewed for lesion volume (skewness: 1.49 to 1.86; kurtosis: 3.00 to 3.69). Accordingly, linear associations are illustrated with the Spearman's rank-order correlation (*r*_s_) for lesions and with Pearson's correlation coefficient (*r*) for normally distributed data.

Attrition (Table 2) was assessed through the comparison of returnees and dropouts at baseline with independent sample *t* tests and total selectivity^32^:

      
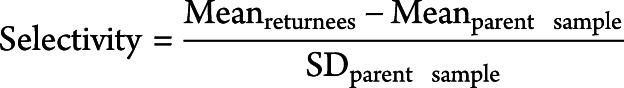


**Table 2:**
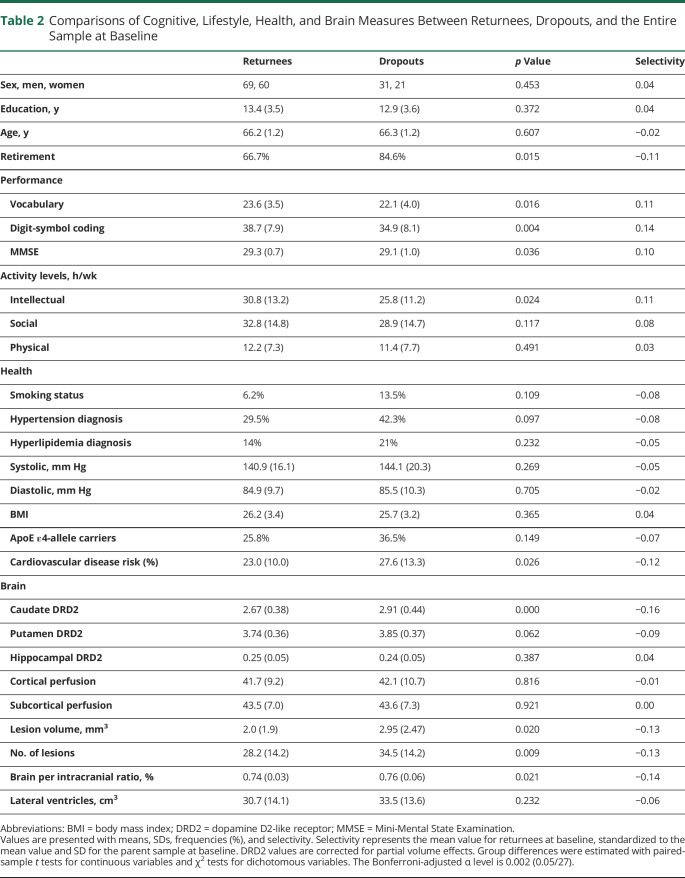
Comparisons of Cognitive, Lifestyle, Health, and Brain Measures Between Returnees, Dropouts, and the Entire Sample at Baseline

Change (%) for DRD2 and structural measures (Table 1) was calculated as:

      
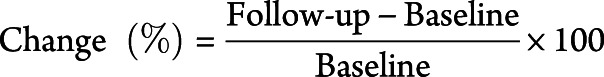


For lesions, change is presented as differences (follow-up minus baseline) because of large variation in baseline levels.

**Figure 1:**
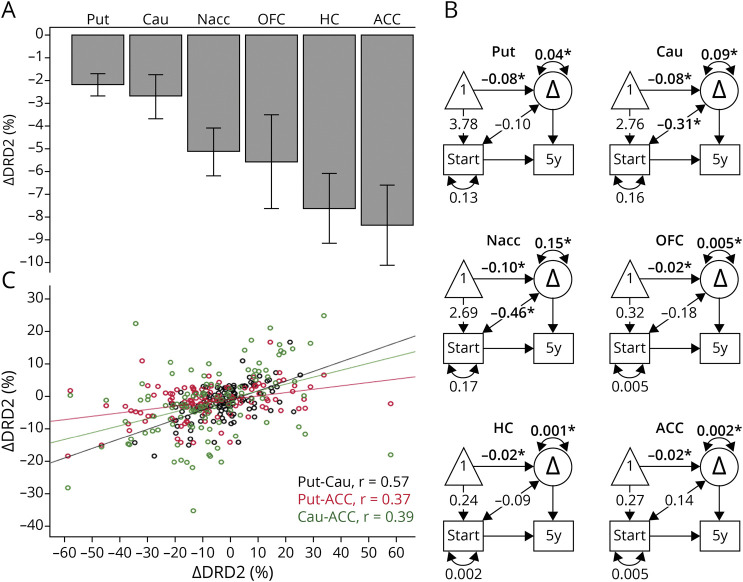
Five-Year Changes for In Vivo DRD2 Availability in Older Adults Significant change was found in the striatum, orbitofrontal cortex, hippocampus, and anterior cingulate cortex (A), with individual differences in rates of change (B). DRD2 changes correlated within the striatum and between striatal and select extrastriatal regions (C). *p < 0.05. In (B), observed variables are represented by a rectangle, a constant by a triangle, and change by a circle. One-sided arrows from constant to baseline and from constant to change represent mean levels at baseline and mean change, respectively. The 2-sided arrows between baseline and change represent correlations, and the 2-sided arrows above the change variables denote individual differences in change. Δ = change; ACC = anterior cingulate cortex; Cau = caudate; DRD2 = dopamine D2 receptor; HC = hippocampus; Nacc = nucleus accumbens; OFC = orbitofrontal cortex; Put = putamen; *r* = Pearson's correlation coefficient.

Paired-sample *t* tests (Table 1, Figure 1A) and repeated-measures analysis of variance (ANOVA) with select covariates (e.g., GM volume on DRD2 change) were conducted to assess statistical significance for brain changes over 5 years and hemispheric differences in change. Regional differences in the magnitude of DRD2 change, and DRD2 change as a function of age, sex, and education, were assessed with univariate or multivariate ANOVA and multivariate regressions. Multiple group comparisons were assessed through Bonferroni post hoc tests.

**Figure 2:**
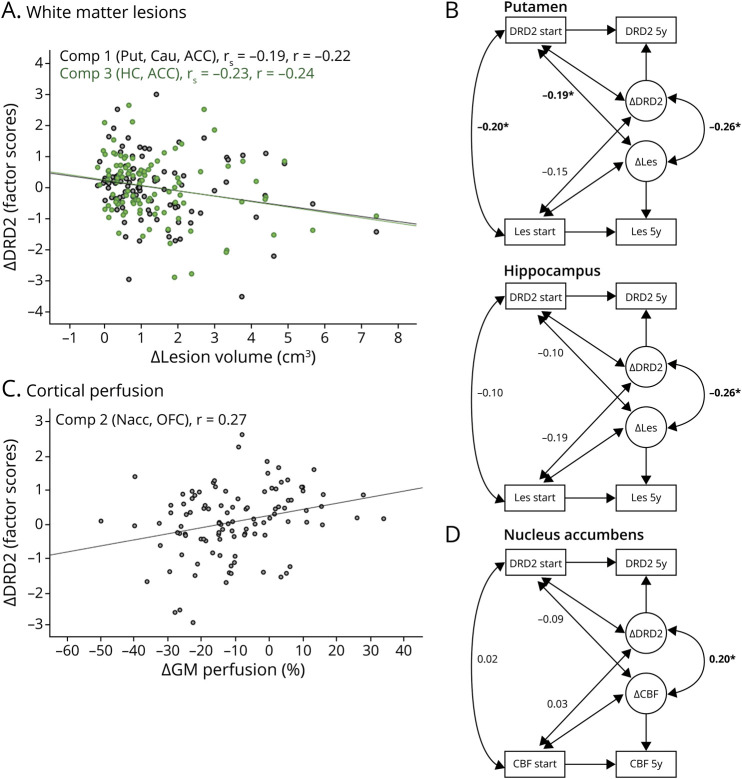
Cerebrovascular Changes Are Associated With DRD2 Decline Increased white matter lesion burden was associated with exacerbated DRD2 decline in the dorsal striatum, hippocampus, and anterior cingulate cortex (A). Bivariate difference score models revealed change-change associations for the putamen and hippocampus (B). Reductions in cortical perfusion were associated with DRD2 losses in the nucleus accumbens and orbitofrontal cortex (C). Change-change links were especially evident for the nucleus accumbens (D). In (B) and (D), observed variables are represented by a rectangle and change by a circle. The 2-sided arrows represent correlations. Note that mean values have been omitted for visual clarity. *p < 0.05. Δ = change; ACC = anterior cingulate cortex; Cau = caudate; CBF = cerebral blood flow; Comp = component; DRD2 = dopamine D2 receptor availability; GM = gray matter; HC = hippocampus; Les = lesions; Nacc = nucleus accumbens; OFC = orbitofrontal cortex; Put = putamen; *r*_s_ = Spearman's rank-order correlation coefficient; *r* = Pearson's correlation coefficient.

Univariate and bivariate difference score models were estimated with the Ωnyx software to assess individual differences in change and for tests of baseline-change and change-change associations (Figure 1B and Figure 2, B and D). Baseline data were entered for the full sample to increase the precision of the mean and variance at the first measurement occasion. In the graphical representations of these models, observed (measured) variables are represented by a rectangle, a constant by a triangle, and change by a circle. The obtained parameters include mean levels at baseline (1-sided arrow from constant to baseline), mean change (1-sided arrow from constant to change variable), individual differences in change (2-sided arrow above change variable), and standardized covariances between baseline DRD2 and change. *Z* values >1.96 or <−1.96 indicate statistical significance at an alpha level of *p* < 0.05. The models reported here are re-expressions of simple difference scores, that is, saturated models that always yield perfect fit to the data. This is why we do not report measures of goodness of fit.

Significant variance in DRD2 change was tested by a 2-df likelihood-ratio test between the original model (Figure 1B) and a corresponding null model, in which the variance for the change variable and its covariance with baseline was set to zero. The interpretation of a significant likelihood-ratio test (*p* < 0.001) is that the variance in change and the covariance cannot be assumed to be zero without losing model fit, hence indicating statistically significant individual differences in rates of change.^33^

Regions with significant mean DRD2 change and individual differences in change (i.e., regions in Figure 1, A and B) were entered into a principal component analysis (PCA) to analyze the patterns of interregional DRD2 change (Table 3). An oblique rotation method (oblimin with Kaiser normalization) was used under the assumption that DRD2 components may be correlated.^19,20^ Components with eigenvalues >1 are reported. With regard to the sample size, standardized loadings >0.50 are considered significant. Factor scores were exported for the DRD2 components and used as dependent variables in multivariate regression analyses, with vascular parameters as independent variables (Figure 2, A and C). The slight skewness of lesion volume data did not affect lesion-DRD2 associations, and a comparable correlation coefficient was achieved with parametric and nonparametric tests (Pearson vs Spearman; Figure 2A). The analysis of cortical vs subcortical perfusion was motivated by observations of aging-related perfusion reductions being higher in several frontal and temporal regions as compared to subcortical regions^34^ and may thus have different bearings on DRD2 losses in these regions. Next, bivariate difference score models were estimated to assess the vascular-DRD2 association at the indicator level (i.e., per DRD2 region; Figure 2, B and D). The models included associations between vascular parameters and DRD2 at baseline, change-change correlations, and standardized covariances between baseline levels in variable a and change in variable b (and vice versa).^35^

**Table 3:**
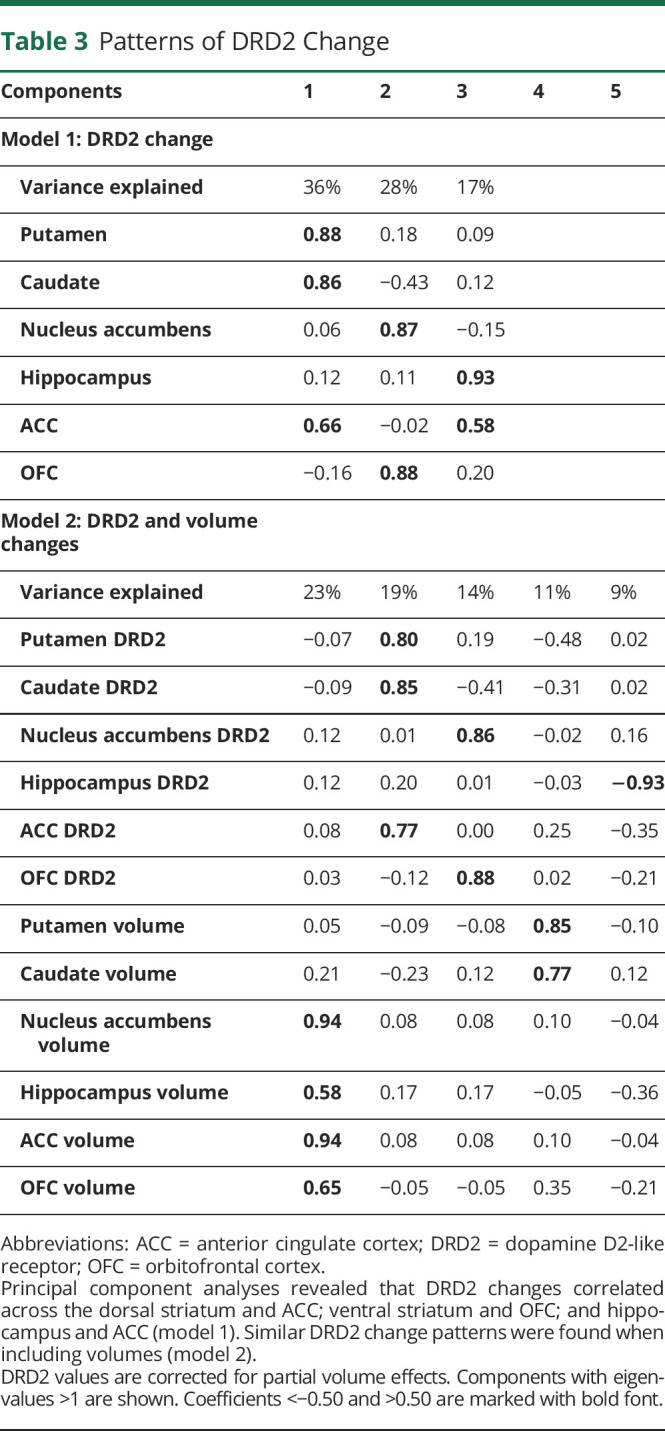
Patterns of DRD2 Change

### Data Availability

The anonymized datasets generated and analyzed during the current study are available from the corresponding author on reasonable request from a qualified investigator. Prerequisites encompass approval of a formal project outline and data sharing agreement and ethical permission for the outlined research questions.

## Results

### Attrition and Longitudinal Selectivity

To assess the degree of nonrandom dropout as a possible threat to generalization,^32^ we investigated longitudinal selectivity. Selectivity was relatively low (0.10–0.16 SDs; Table 2). Participants not returning for the follow-up session were somewhat more likely to be retired, to show lower cognitive baseline performance and activity levels, having poorer vascular health, and having higher caudate DRD2 levels at baseline. Three individuals died between test waves. Elevated caudate DRD2s for dropouts reflect high dropout (43%) from a previously identified subgroup characterized by low cognition but high DRD2 levels.^29^ Dropout was 21% for a subgroup with high cognition and high DRD2 levels and 33% for a group with low cognition and low DRD2 levels (*p* = 0.032; eFigure 1, links.lww.com/WNL/C161).

For the returnees, significant 5-year changes included reduced systolic (140.9 ± 16.1 and 136.5 ± 17.2, *t*(128) = 2.9, *p* = 0.004) and diastolic (84.9 ± 9.7 and 82.4 ± 10.1, *t*(127) = 2.9, *p* = 0.005) blood pressure, likely reflecting additional medications at follow-up (number of medicines: 1.26 ± 1.7 at baseline and 2.0 ± 1.9 at follow-up; *t*(128) = 3.2, *p* = 0.001). The frequency of medication increased from 30% to 46% for hypertension and from 14% to 29% for hyperlipidemia. The BMI remained unchanged over time (26.1 ± 3.3 and 26.3 ± 3.6, *t*(126) = −1.2, *p* = 0.225). Five participants were diagnosed with diabetes mellitus between waves, which was an exclusion criterion at baseline but not at follow-up. No cases of neurologic disorders (e.g., Parkinson disease) were noted at follow-up. The frequency of retirement was 67% at baseline and 91% at follow-up.

### Five-Year Changes in DRD2 Availability

The PVE-corrected DRD2 measures revealed significant 5-year DRD2 reductions within the striatum (putamen: *t*(118) = 4.49, caudate: *t*(118) = 2.47, and nucleus accumbens: *t*(118) = 3.67; Table 1 and Figure 1A) and also in select extrastriatal regions (anterior cingulate cortex [ACC]: *t*(116) = 4.89, hippocampus: *t*(118) = 5.33, and orbitofrontal cortex [OFC]: *t*(117) = 3.10). Difference score models confirmed significant individual differences in DRD2 change for these regions (see double-headed arrow above the change factor; Figure 1B). The magnitude of DRD2 loss (%) was comparable among striatal regions but higher extrastriatally (*F*(5, 740) = 3.4, *p* = 0.004; Figure 1A). Specifically, between-region comparisons revealed that DRD2 decline was significantly larger in the ACC, as compared to the putamen (*p* = 0.014) and caudate (*p* = 0.045). Furthermore, significant DRD2 reductions remained after adjustment for GM loss in each region (*F* values with/without covariates: putamen: 40.2/20.1, caudate: 10.7/6.1, nucleus accumbens: 7.1/13.4, hippocampus: 8.9/28.4, ACC: 18.8/20.1, and OFC: 8.8/9.6; *p* < 0.05 for all). It should be noted that a significant DRD2 decline was found within these regions also when analyzing DRD2 BP_ND_ values derived from the pipeline without PVE correction (without PVC in Table 1). Reliable DRD2 change was not observed in other extrastriatal regions (eTable 2, links.lww.com/WNL/C161).

Within-person regional DRD2 change did not differ across hemispheres (*t*(111–117) = 0.2–1.1; *p* > 0.05), nor as a function of age (*F*(6, 104) = 0.9, *p* = 0.523) or level of educational attainment (*F*(6, 104) = 1.0, *p* = 0.446). We have previously reported sex differences for striatal DRD2 levels at baseline.^18,36^ No sex differences were found for DRD2 decline in the dorsal striatum or in the ACC, hippocampus, or OFC (*p* > 0.05); however, women showed steeper DRD2 decline rates than men in the nucleus accumbens (−8.0% vs −3.0%; *F*(1,110) = 5.8, *p* = 0.018). Analyses with values derived from the pipeline without PVE correction revealed no association between DRD2 change in relation to age (*F*(6, 116) = 1.2, *p* = 0.304), level of educational attainment (*F*(6, 116) = 0.7, *p* = 0.624), or sex (*F*(6,116) = 2.0, *p* = 0.071). However, exacerbated right-sided DRD2 reductions were found for the nucleus accumbens (*t*(125) = 3.2, *p* = 0.002), OFC (*t*(125) = 5.0, *p* < 0.001), and hippocampus (*t*(125) = 2.1, *p* = 0.041).

### Patterns of Striatal and Extrastriatal DRD2 Changes

A PCA was conducted to investigate the structure of regionally correlated DRD2 changes across those regions showing a significant mean change and variance in change (i.e., the regions in Figure 1, A and B). Three principal components were significant in a rotated solution (model 1 in Table 3). The first component (36% of variance explained) included the caudate, putamen, and ACC. The nucleus accumbens and OFC loaded strongly on the second component (28% of variance), whereas the hippocampus and ACC loaded onto a third component (17% of variance). Pairwise correlations for DRD2 change in regions loading onto the first component are illustrated in Figure 1C. However, no between-component correlations were found (component 1 vs 2: *r* = −0.06, *p* = 0.546; 1 vs 3: *r* = 0.16, *p* = 0.092; 2 vs 3: *r* = 0.01, *p* = 0.943), indicating the presence of distinct patterns of DRD2 change. A similar pattern was found when analyzing the BP_ND_ values without PVE correction (eTable 1, links.lww.com/WNL/C161).

The potential influence of GM atrophy to DRD2 change was assessed through a second PCA where DRD2 change and volume change per ROI was entered. This model demonstrated correlations between ACC-striatal, as compared to ACC-hippocampal, DRD2 changes (Table 3, model 2). Furthermore, correlations were found for volume changes and DRD2 change for the dorsal striatum. Significant zero-order correlations for DRD2 vs volume change were found for the striatum across both DRD2 pipelines, but not for extrastriatal regions (eTable 3, links.lww.com/WNL/C161).

### Relationships Between Cerebrovascular Health and DRD2 Decline

Sizable perfusion reductions were observed across 5 years (approx. −6 to −10%; Table 1). WM lesion burden varied greatly, and individuals with the largest lesion diameters at baseline demonstrated larger 5-year increases in lesion burden (0.5 ± 0.5 cm^3^, 1.41 ± 1.1 cm^3^, and 3.9 ± 2.0 cm^3^ for individuals with lesion diameters of <10, 10–20, and >20 mm at baseline, respectively; *F*(2, 118) = 43.7, *p* < 0.001). GM perfusion was negatively associated with WM lesion burden at baseline (cortical perfusion: *r*_*s*_ = −0.22, *p* = 0.019; subcortical perfusion: *r*_*s*_ = −0.17, *p* = 0.061). However, no change-change associations were found for these variables (*r*_*s*_ = 0.08 and 0.09 for cortical and subcortical perfusion, respectively; *p* values >0.05).

No association was found among WM lesion volume and cortical or subcortical GM volume at baseline (*r*'s = 0.15 and 0.11, respectively, *p*'s > 0.05). WM lesion volume change was associated with cortical (*r* = −0.21, *p* = 0.026), but not subcortical (*r*'s = 0.12, *p* > 0.05) volume change. No perfusion-volume associations were found for cortical (*r* = −0.03, *p* > 0.05) or subcortical GM (*r* = −0.02, *p* > 0.05) at baseline or for changes among these measures (*r*'s = 0.09 and −0.05, *p*'s > 0.05).

Factor scores were extracted for the 3 distinct dimensions of DRD2 losses (Table 3) and entered as dependent variables in a multivariate regression analysis with (1) change in WM lesion volume, (2) change in frontotemporal GM perfusion, and (3) subcortical GM perfusion as independent variables. DRD2 change was associated with changes in lesion volume (*F*(3,94) = 3.7, *p* = 0.015) and cortical perfusion (*F*(3,94) = 3.3, *p* = 0.024). Specifically, increases in WM lesion volume were associated with more pronounced DRD2 loss in regions of component 1 (caudate, putamen, and ACC; *F*(1,96) = 5.1, *p* = 0.026) and component 3 (hippocampus and ACC; *F*(1,96) = 6.0, *p* = 0.016; Figure 2A), whereas reduced frontotemporal GM perfusion was associated with DRD2 loss in regions of component 2 (nucleus accumbens and OFC; *F*(1,96) = 8.1, *p* = 0.006; Figure 2C).

Bivariate difference score models demonstrated that baseline lesion burden was associated with baseline DRD2 levels in the putamen (Figure 2B), but also in the caudate (*r* = −0.34, *p* < 0.05) and ACC (*r* = −0.28, *p* < 0.05). Significant change-change associations were found for lesions and DRD2 levels in the putamen and hippocampus (Figure 2B). Moreover, baseline DRD2 levels in the putamen (Figure 2B), caudate (*r* = −0.26, *p* < 0.05), and ACC (*r* = −0.18, *p* < 0.05) were associated with change in lesion burden. Change-change associations were found for cortical GM perfusion and DRD2 in the nucleus accumbens (Figure 2D), but not the OFC (*r* = 0.14).

## Discussion

This work provides the first longitudinal evidence for aging-related DRD2 losses and paves the way for a mechanistic understanding for such reductions. The magnitude of striatal DRD2 reductions was about half of previous cross-sectional estimates of age differences across the adult lifespan (∼4% vs ∼8% per decade).^4^ This demonstrates once again differences in cross-sectional approximations vs true longitudinal measurements of age-related changes. Thus, previous statements about the magnitude of average DA decline,^3,4^ at least for the age segment of ∼65–75 years, need to be revised. Cohort effects and infrequent use of PVE correction may have contributed to the higher magnitude of the cross-sectional estimates.^25^ For instance, caudate DRD2 reductions were twice as large for uncorrected, as compared to PVE-corrected, values. DRD2 losses correlated across striatal and select extrastriatal regions and were associated with changes in cerebrovascular parameters. DRD2 losses were primarily observed in regions particularly susceptible to vascular insults, such as basal ganglia and hippocampus.^21,37^

The current study demonstrates that within-person DRD2 changes in aging are particularly marked in several associative and limbic regions, such as caudate, nucleus accumbens, hippocampus, and ACC. These are also regions with prominent dopaminergic losses when cognitive impairments and dementia emerge.^38^ Cross-sectional studies have revealed mixed regional rank orders for age differences in DRD2s. Using ^18^F-fallypride/PET, the largest age differences were demonstrated for frontal and temporal DRD2s (−6% to −16% per decade), followed by striatal (−3% to −5%) and hippocampal DRD2s (0% to −2%).^39^ ^11^C-FLB 457/PET revealed similar cortical and hippocampal DRD2 reductions as reported in the current study (−12% to −14% per decade).^40^ Insufficient statistical power, cohort effects, and methodologic choices (e.g., ligand characteristics) may underlie the diverging age effects on hippocampal DRD2s in previous studies.^12,25,39^

Atrophy can influence ligand-binding estimates, with overestimated aging effects as a result.^25^ Notably, analyses on PVE-corrected and uncorrected DRD2 values revealed similar regional patterns, with lower DRD2 reductions in a few regions after PVE correction. The patterns of change remained after controlling for within-person atrophy, underscoring that DRD2 decline was not driven by volume losses. This is in line with experimental work conducted at the same scanner and with the same reconstruction methods.^41^ Other sources of variation across DRD2 studies are differences in ligand characteristics. Despite low accumulation and a signal-to-noise ratio in extrastriatal regions,^42^ a growing number of studies demonstrate reliability and validity for extrastriatal ^11^C-raclopride binding.^11,20,43,44^

There is a distinct topography of midbrain DA neuron projections.^45,46^ DRD2 losses were correlated within DA pathways, as indicated previously,^19,20,47^ but also between DA pathways (striatum-ACC). The distinct pattern of DRD2 decline in interconnected structures of the reward circuit (ventral striatum and OFC),^46^ and across associative and motor regions (dorsal striatum, hippocampus, and ACC), with links to reduced frontotemporal perfusion and lesion progression, respectively, is noteworthy. The relationship between cerebral perfusion and lesion manifestation in aging is unclear.^48^ Perfusion data, as quantified with pcASL, have a fair amount of noise, which was minimized here through averaging of voxels in relatively large ROIs. The distinct links between dorsal vs ventral striatal DRD2 levels and the vascular parameters may stem from separate microvascular territories within the striatum, giving rise to differential susceptibility to ischemic events.^49^ Another factor could be proximity to the epicenter where vascular injury typically manifests.

A conceivable mechanism that might explain the observed associations is that vascular dysfunction exerts insult in target regions of DA nerve fibers. Still, it is not possible to firmly delineate the direction of causality between vascular and DA alterations. As shown here, baseline DRD2 status was associated with lesion progression. There is a vasomotor response after provision of DA agonists,^50^ and patients with Parkinson disease are at higher risk for cardiovascular disease.^16^ Hence, the relationship between these cascades may be reciprocal. Generalizability of our findings is limited by the narrow age range of the COBRA sample, and that dropout may have led to selection bias. Consequently, we cannot exclude the possibility that DRD2 decline rates may differ in younger vs older age segments. Furthermore, the DRD2-vascular link may be underestimated in older persons because compromised vascular health predicted dropout. DRD2 reductions in younger age segments (30 years and onward^4^) may be attributable to factors other than vascular health.

This longitudinal study provides new knowledge on how aging affects DRD2 levels and suggests that individual differences in rates of DRD2 change are related to vascular health. Future work may consider conducting extensive mapping of several cerebrovascular events^48^ to further examine the DA-vascular link in aging.

## Glossary

ACC: anterior cingulate cortex
ANOVA: analysis of variance
BMI: body mass index
BP_ND_: DRD2 binding potential
COBRA: Cognition, Brain, and Aging
DA: dopamine
DRD2: DA D2-like receptor
GM: gray matter
MMSE: Mini-Mental State Examination
MRTM: multilinear reference tissue model
OFC: orbitofrontal cortex
PCA: principal component analysis
pcASL: pseudocontinuous arterial spin labeling
PVE: partial volume effect
ROI: region of interest
WM: white matter
